# Emerging theory of sensitization in post-stroke muscle spasticity

**DOI:** 10.3389/fresc.2023.1169087

**Published:** 2023-09-15

**Authors:** Areerat Suputtitada

**Affiliations:** Department of Rehabilitation Medicine, Faculty of Medicine, Chulalongkorn University, and King Chulalongkorn Memorial Hospital, The Thai Red Cross Society, Bangkok, Thailand

**Keywords:** muscle spasticity, extracorporeal shockwave therapy, ESWT, peripheral magnetic stimulation, PMS, needling, mechanical needling and sterile water injection, sensitization

## Abstract

Spasticity, characterized by a velocity-dependent increase in muscle tone and exaggerated reflexes, is a common complication in individuals with upper motor neuron syndrome, such as stroke survivors. Sensitization, the heightened responsiveness of the nervous system to sensory stimuli, has emerged as a potential cause of spasticity. This perspective article explores three emerging treatments targeting sensitization. Recent studies have investigated novel treatment modalities for spasticity, including Extracorporeal Shockwave Therapy (ESWT), repetitive peripheral magnetic stimulation (rPMS), and needling. ESWT has shown promising results in reducing spasticity in both the upper and lower extremities, potentially through mechanisms such as nitric oxide production, rheological property changes, and neuromuscular transmission dysfunction. rPMS offers a non-invasive approach that may reduce spasticity by increasing sensory input, enhancing cortical activation, and exerting tissue-softening effects. Needling has also demonstrated positive effects on spasticity reduction. The high heterogeneity observed indicates the need for more rigorous research to confirm these findings. Recently, mechanical needling and sterile water injection invented by the author is also promising for reducing spasticity through removing sensitization. In conclusion, the emerging treatment options discussed in this perspective article provide promising avenues for addressing sensitization in spasticity and improving motor function. However, further research is needed to validate their findings, optimize treatment protocols, and investigate their long-term effects on motor recovery and overall quality of life in individuals with spasticity.

## Introduction

Spasticity, characterized by increased muscle tone and exaggerated tendon jerks, is a common complication of stroke and primary motor impairment (1). It is caused by stretch reflex hyperexcitability, which is a symptom of upper motor neuron (UMN) syndrome. The lack of agreement on its definition, on the other hand, highlights the complexity and diversity of spasticity (2).

In people with UMN syndrome, weakness reduces mobilization of affected muscles, exacerbating spasticity. Muscle immobilization also impedes post-activation depression, a critical mechanism in the development of spasticity (3). The interaction of spasticity and weakness causes contractures, abnormal joint postures, and limited movement, all of which have a significant impact on daily activities. Mechanical and morphological changes in intracellular and extracellular components, as well as structural changes in muscle and tendon fibers, all play a role in the onset and progression of spasticity (3–5). Spasticity is associated with muscle fiber lengthening and a decrease in sarcomere count (3–5).

Sensitization may be a significant cause of spasticity, according to new research. Extracorporeal Shockwave Therapy (ESWT) has shown promise as an adjunct therapy to improve motor recovery after botulinum toxin injections (6). ESWT has been shown in meta-analyses of randomized controlled trials in stroke patients to be effective in reducing upper-limb spasticity for more than 12 weeks (7, 8) and lower-extremity spasticity for more than 4 weeks (7, 9). ESWT is preferred over botulinum toxin because it is non-drug and does not cause neuromuscular denervation. However, more research is needed to investigate different modes of ESWT application and compare its efficacy to that of botulinum toxin.

Furthermore, repetitive peripheral magnetic stimulation (rPMS) has the potential to help with post-stroke spasticity, but optimal treatment protocols and durations have yet to be determined (10). When used in conjunction with rehabilitation therapy, needling has been shown to be effective in reducing post-stroke spasticity in the lower extremities after one week but loses efficacy after 4 weeks (11).

The purpose of this perspective article is to explore the potential mechanisms underlying these emerging treatments and evaluate the relationship between peripheral sensitization and muscle spasticity. By better understanding these mechanisms, we can develop better therapeutic strategies to manage spasticity and improve the daily lives of patients who suffer from it.

## Sensitization is possible as a cause of spasticity

Spasticity is an early manifestation of maladaptive plasticity that can persist through the chronic phase. It indicates that spasticity is closely associated with other motor impairments, such as abnormal force control, muscle coactivation, motor synergies, and diffuse interlimb muscle activation. There are two main mechanisms that cause spasticity after a stroke: reflexively mediated mechanisms and non-reflexively mediated mechanisms. Increased stretch reflexes characterize the reflexively mediated mechanism of spasticity (12–15). Damage to the motor cortex and its corticospinal descending tract causes immediate hemiplegia, affecting the muscles of the affected upper and lower limbs as well as, to a lesser extent, the trunk muscles. Damage to the corticobulbar pathway, as well as the motor cortex or corticospinal descending tract, results in a loss of supraspinal inhibition and bulbar spinal cord overexcitation. Because of the unopposed excitatory descending inputs from the reticulospinal tract, this de-inhibition phenomenon, or de-masking effect, leads to increased excitability of spinal motor neurons (12–15). The occurrence of damage to the cerebral cortex can lead to a disruption in the inhibitory control exerted by lower motor neurons over upper motor neurons, resulting in a state of heightened excitation (16). This adaptive change can explain a variety of clinical signs of spasticity, including hyperreflexia, velocity-dependent resistance to stretch, excessive muscle activity, and spontaneous motor unit discharge.

Spasticity following stroke is associated with changes in the mechanical properties of muscles, tendons, joints, and other tissues, which are part of the non-reflexively mediated mechanism. The focus on spasticity following stroke has primarily been on brain injury, with less attention paid to muscle tissue structure, metabolism, and function. However, spasticity causes changes in muscle function and structure, including changes in muscle fiber size, fiber type distribution, extracellular matrix proliferation, and increased stiffness of spastic muscle cells. Spastic muscles have poorer extracellular mechanical properties than normal muscles (12–18). After stroke, changes in muscle fiber characteristics, proportion, and length contribute to changes in the physiological function and biomechanical characteristics of affected skeletal muscles, exacerbating the spastic state (12–18).

A new definition of poststroke spasticity and the interference of spasticity with motor recovery from acute to chronic stages highlights that atypical neuroplasticity following a stroke leads to spasticity and related motor issues (18). While spasticity might not affect immediate functional recovery, it does impede “true” motor improvement by causing abnormal movement patterns and muscle weakness. Factors like abnormal force control, muscle coactivation, and interlimb coupling further complicate recovery. Managing spasticity involves strategies like realigning mechanics and neuromuscular reeducation. Although reducing spasticity may not directly correlate with functional improvement, effectively addressing it can enhance motor function during the chronic stroke phase. Understanding this interaction is crucial for optimizing rehabilitation efforts.

In conclusion, both reflexively and non-reflexively mediated mechanisms, such as changes in muscle tissue structure and mechanical properties brought on by cortical and supraspinal changes, are responsible for spasticity after stroke (12–18).

The process by which the nervous system becomes more responsive to sensory stimuli, resulting in an exaggerated response, is referred to as sensitization in muscle spasticity. Sensitization in the context of muscle spasticity refers to increased sensitivity of the muscle spindles and other sensory receptors in the muscles and tendons, which results in increased muscle tone, involuntary muscle contractions, and exaggerated reflexes. The mechanisms underlying sensitization in muscle spasticity involve complex interactions between the nervous system's sensory and motor pathways. The release of neurotransmitters, particularly glutamate and substance P, which play a role in the transmission of pain signals and the regulation of muscle tone, is an important factor (13, 18–20). Sensitization can result from repeated or prolonged activation of sensory receptors, such as muscle spindles, caused by factors such as muscle stretch, inflammation, or injury. This prolonged activation can alter neuron excitability in the spinal cord and brain, resulting in an amplified response to subsequent sensory input. Sensitization can result in a feedback loop in which increased muscle tone and reflexes contribute to more sensory input, exacerbating the condition. Understanding the role of sensitization in muscle spasticity is important for developing effective treatment strategies. By targeting the mechanisms involved in sensitization, such as modulating neurotransmitter release or reducing sensory input, it may be possible to alleviate spasticity and improve motor function in individuals with spasticity after a stroke or other neurological conditions.

## ESWT for muscle spasticity and sensitization theory

In 2022, Zhang et al. (7) conducted a systematic review and meta-analysis to evaluate the efficacy of ESWT on spasticity after upper motor neuron injury. The review included 42 studies with a total of 1973 patients, and the meta-analysis included 34 studies. There were 29 studies on stroke patients, and others on patients with multiple sclerosis (MS), spinal cord injury (SCI), and cerebral palsy (CP). The results of the subgroup analysis suggested that radial ESWT was more effective in relieving spasticity than focused ESWT, possibly due to its larger therapeutic area and higher energy application to superficial tissue. Higher pressure and frequency (6 Hz) were linked to better results. The effects of ESWT were found to last for a month after treatment. A single session of ESWT, on the other hand, had no significant effect on the Modified Ashworth Scale (MAS), which is commonly used to assess muscle spasticity. Because of the lack of blinding of patients and interventionists, which is a limitation of this type of treatment, the authors acknowledged a potential risk of bias in the included studies.

The effects of ESWT on spasticity are thought to involve multiple mechanisms, including nitric oxide production, rheological property changes, motor neuron excitability reduction, and induction of neuromuscular transmission dysfunction (6–8, 16, 21).

The induction of nitric oxide (NO) synthesis is one proposed mechanism. ESWT may stimulate the production of nitric oxide, which is involved in the formation of neuromuscular junctions and has a variety of physiological functions. Nitric oxide synthesis may contribute to ESWT's therapeutic effects by promoting neovascularization (22, 23), regulating inflammation and suppression of leukocyte formation (24, 25), and activating growth factors in spastic muscle, intensification of tissue regeneration, and decrease tissue apoptosis (26). Nitric oxide is known to have various physiological functions in the CNS, including neurotransmission and neuroprotection (27).

Animal experiments have also shown regenerative properties of ESWT in the CNS. ESWT may induce a partial destructive impact that removes degenerated axons, followed by an increase in the ability to create new axons. This phenomenon promotes axonal regeneration and may contribute to functional recovery (28).

ESWT has been found to enhance the neuroprotective effect of vascular endothelial growth factor (VEGF) (29, 30). VEGF plays a crucial role in promoting angiogenesis and neuroprotection (29, 30). By enhancing the effects of VEGF, ESWT may improve neurological function and support tissue repair in CNS diseases. Furthermore, ESWT has been shown to affect the expression of neurotrophins, such as neurotrophin-3 (NTH-3), which promote the survival and regeneration of neurons (31). ESWT can increase the expression of NTH-3, and repeated applications of ESWT can stimulate the activity of macrophages and Schwann cells. These cellular responses contribute to the survival and regeneration of neurons (31).

According to the experimental data report, Schwann cells treated with ESWT showed significant improvements in isolation, culture, and proliferative capacities. This finding is clinically significant, especially in the context of peripheral nervous system damage (32). The improved ability to isolate, culture, and proliferate Schwann cells may help develop therapeutic interventions for peripheral nerve injuries.

Furthermore, the findings suggest that ESWT causes reversible segmental demyelination of large-diameter nerve fibers. However, this demyelination had no significant negative impact on their performance (32). Because the reversible demyelination did not result in functional impairment, this suggests that ESWT may be a viable treatment option. The studies have shown that ESWT is effective in improving peripheral nerve function and can help reduce denervation atrophy. This suggests that ESWT has the potential to treat peripheral nerve injuries by promoting nerve regeneration and preventing denervation-related muscle wasting (32, 33).

Mechanical shock or vibration caused by ESWT has an immediate impact on the rheological properties of spastic muscles. This effect may disrupt the functional link between actin and myosin, causing the connective tissue within the spastic muscle to loosen. ESWT may also cause vasodilation via enzymatic and non-enzymatic nitric oxide synthesis, increasing blood flow to the area and influencing the secretion of interleukins involved in inflammation and growth factor activation (6–8, 16, 21).

By applying pressure to tendons, ESWT may also reduce motor neuron excitability. This decrease in excitability of motor neurons can help alleviate spasticity and improve motor function. ESWT has also been shown to affect myofascial viscoelasticity, muscle stiffness, and connective tissue, all of which contribute to the reduction of non-neuronal aspects of spasticity. ESWT has also been shown to be effective in treating calcification in tendons and joints and breaking down fibrosis in chronic muscle spasticity (6–8, 16).

Another mechanism by which ESWT may be effective is by causing neuromuscular transmission dysfunction. ESWT has been shown in studies to reduce acetylcholine receptors in rabbit neuromuscular junctions, potentially affecting neuromuscular junction function and spasticity (6–8, 16, 21).

In a small number of studies, the safety profile of ESWT revealed minimal adverse effects, including skin injury, bone distortion, muscle numbness, pain, petechiae, and weakness (6–8).

Overall, ESWT has shown promising results in stroke patients in terms of relieving spasticity, reducing pain, and improving range of motion and motor function. While the meta-analysis suggests that ESWT may be effective and safe for treating spasticity after upper motor neuron injury, the limitations of the included studies and the high heterogeneity observed indicate the need for more rigorous research to confirm these findings (34, 35). In addition, more research is needed to fully understand the underlying mechanisms and potential long-term benefits of this therapy.

## rPMS for muscle spasticity and sensitization theory

According to a systematic review and meta-analysis by Pan et al. in 2022 (10) of eight randomized controlled trials (RCTs) involving 170 patients with chronic stroke, repetitive peripheral magnetic stimulation (rPMS) showed potential for reducing spasticity in both the upper and lower extremities.

The exact mechanism by which rPMS decreases spasticity is not fully understood, but it is believed to involve a combination of increased somatosensory and proprioceptive input, cortical activation, and tissue softening effects (10, 36, 37). Studies have shown that sensory deficits are associated with the development of spasticity, and rPMS may help increase somatosensory and proprioceptive afferents. rPMS can directly activate sensorimotor nerve fibers and indirectly activate mechanoreceptors during muscle contraction, relaxation, and vibration. This increased proprioception and somatosensation may contribute to reducing spasticity. Furthermore, rPMS may enhance corticomotor excitability through the structural and functional connections between the sensory and motor cortices. This increased corticomotor excitability can lead to increased inhibitory regulation of the stretch reflex, thereby reducing spasticity (10, 36, 37).

Another potential mechanism of rPMS in spasticity reduction relates to its local effects on the tissues. Studies have shown that rPMS can decrease muscle hardness and increase blood flow to the muscle. The tissue-softening effect of rPMS may contribute to the reduction of spasticity (10, 36, 37).

A study (38) revealed that repeated sessions of piTBS (paired associative theta burst stimulation) applied to spastic muscles have been found effective in decreasing spasticity, even in cases of higher grades. This reduction in spasticity may subsequently lead to a decrease in the required dose of Botulinum toxin for injection, which is commonly used to manage spasticity. The statement also recommends further studies to explore the impact of these positive effects on function and their long-term effects. If proven to be effective, piTBS could offer advantages over standard high-frequency protocols by consuming less time while maintaining treatment efficacy. This suggests that piTBS may provide a more efficient and time-saving approach to managing spasticity.

However, there are limitations to the interpretation of the results of studies on rPMS and spasticity reduction. The participants in the studies had varying levels of spasticity severity, and the effects of rPMS on spasticity reduction could not be specific to the degree of spasticity. Additionally, the outcome measurements used in the studies may not fully address reflex-mediated stiffness, and future studies could benefit from more reliable and reproducible spasticity tests. The evidence obtained from meta-analyses based on small sample sizes is not robust, and further studies with consistent rPMS parameters are needed to confirm the effect size for each outcome. Additionally, the optimal protocol for rPMS in clinical practice for spasticity treatment, including parameters such as frequency, intensity, coil type, number of pulses, and duty cycle, still needs to be determined.

Overall, rPMS studies show promise as an intervention for spasticity and motor function impairment due to central nervous system (CNS) lesions. However, further research is necessary to fully understand the underlying mechanisms and establish the optimal rPMS protocol for clinical practice in spasticity treatment.

## Needling for muscle spasticity and sensitization theory

The meta-analysis found evidence indicating that dry needling had a positive effect on reducing spasticity (muscle tone) in stroke survivors, with very low to moderate certainty (11). However, no significant effects on motor function were observed. It is important to note that the pooled data did not show significant effects on spasticity in the upper extremity, highlighting the need for further research in this specific area. Most of the trials included in the meta-analysis focused on short-term effects, with a follow-up period of only one week. Only two studies investigated longer follow-up periods of 4–6 weeks (11). Therefore, there is a need for randomized clinical trials that examine the long-term effects of dry needling in post-stroke patients to gain a more comprehensive understanding of its effectiveness.

Regarding the techniques used in previous studies, dry needling was applied over the most painful spot within a spastic, taut band. Active trigger points were targeted to reproduce referred pain, and specific belly muscle points were standardized. Dry needling may achieve an antinociceptive effect by triggering various neurophysiological mechanisms, both peripherally and centrally mediated. Studies have shown that dry needling can increase pressure pain thresholds and induce changes in brain function (11, 13). To further understand the mechanisms and potential benefits of dry needling, more research is required. Recent innovations created by Areerat Suputtitada in mechanical needling and sterile water injection for calcification and fibrosis removal (39, 40) may promise improvements in muscle spasticity. The mechanical needling procedure entails the physical extraction of calcification and fibrosis. The objective of sterile water injection is to augment the disintegration of calcification and fibrosis. Both interventions have the potential to decrease sensitivity and activate pain-blocking pathways while also inducing changes in neurochemistry, with the ultimate objective of mitigating pain (39, 40). This aims to break down fibrosis or calcification in chronic muscle spasticity and may have an enhanced analgesic effect, cause a decrease in muscle sensitization. Mechanical needling has the potential to decrease central and peripheral sensitization and reduce the sources of peripheral nociception, such as trigger points, calcification, and fibrosis (39, 40). The possibility of biomarker changes with these innovations should be investigated in future studies.

Persistent peripheral painful stimulation can lead to a phenomenon called central sensitization, where the central nervous system becomes hypersensitive, amplifying pain signals and contributing to the development of chronic pain. It is crucial to explore and investigate effective treatment options for chronic pain associated with muscle spasticity in light of the sensitization theory.

## Additional challenges

These emerging treatments have shown evidence of effectiveness in reducing pain, which is the primary cause of spasticity. However, it is important to consider the heterogeneity of patients in these studies. Subgroup analysis should be conducted specifically on patients who do not have nociceptive or neuropathic pain. This approach can help identify the most appropriate treatment options for individuals with pain and muscle spasticity, taking into account their specific needs and characteristics.

## Conclusion

Spasticity is a common complication of stroke, and sensitization is believed to be a potential cause of muscle spasticity. Emerging treatments such as rESWT, rPMS, and needling show promise in reducing spasticity and improving motor function. These treatments focus on the underlying causes of sensitization, such as changes in neurotransmitters, more sensory input, softening of tissues, and problems with neuromuscular transmission. However, the high heterogeneity observed indicates the need for more rigorous research to confirm these findings. Additionally, further research is needed to fully understand the mechanisms, long-term benefits, and risks of these therapies.
